# Differential network analysis and protein-protein interaction study reveals active protein modules in glucocorticoid resistance for infant acute lymphoblastic leukemia

**DOI:** 10.1186/s10020-019-0106-1

**Published:** 2019-08-01

**Authors:** Zaynab Mousavian, Abbas Nowzari-Dalini, Yasir Rahmatallah, Ali Masoudi-Nejad

**Affiliations:** 1 School of Mathematics, Statistics, and Computer Science, College of Science, University of Tehran, Tehran, Iran; 2 Laboratory of Systems Biology and Bioinformatics (LBB), Institute of Biochemistry and Biophysics, University of Tehran, Tehran, Iran; 3 Department of Biomedical Informatics, University of Arkansas for Medical Sciences, Little Rock, AR 72205 USA

**Keywords:** Acute lymphoblastic leukemia, Glucocorticoid resistance, Differential co-expression network analysis, Systems biology, Active protein modules

## Abstract

**Background:**

Acute lymphoblastic leukemia (ALL) is the most common type of cancer diagnosed in children and Glucocorticoids (GCs) form an essential component of the standard chemotherapy in most treatment regimens. The category of infant ALL patients carrying a translocation involving the mixed lineage leukemia (MLL) gene (gene KMT2A) is characterized by resistance to GCs and poor clinical outcome. Although some studies examined GC-resistance in infant ALL patients, the understanding of this phenomenon remains limited and impede the efforts to improve prognosis.

**Methods:**

This study integrates differential co-expression (DC) and protein-protein interaction (PPI) networks to find active protein modules associated with GC-resistance in MLL-rearranged infant ALL patients. A network was constructed by linking differentially co-expressed gene pairs between GC-resistance and GC-sensitive samples and later integrated with PPI networks by keeping the links that are also present in the PPI network. The resulting network was decomposed into two sub-networks, specific to each phenotype. Finally, both sub-networks were clustered into modules using weighted gene co-expression network analysis (WGCNA) and further analyzed with functional enrichment analysis.

**Results:**

Through the integration of DC analysis and PPI network, four protein modules were found active under the GC-resistance phenotype but not under the GC-sensitive. Functional enrichment analysis revealed that these modules are related to proteasome, electron transport chain, tRNA-aminoacyl biosynthesis, and peroxisome signaling pathways. These findings are in accordance with previous findings related to GC-resistance in other hematological malignancies such as pediatric ALL.

**Conclusions:**

Differential co-expression analysis is a promising approach to incorporate the dynamic context of gene expression profiles into the well-documented protein interaction networks. The approach allows the detection of relevant protein modules that are highly enriched with DC gene pairs. Functional enrichment analysis of detected protein modules generates new biological hypotheses and may help in explaining the GC-resistance in MLL-rearranged infant ALL patients.

**Electronic supplementary material:**

The online version of this article (10.1186/s10020-019-0106-1) contains supplementary material, which is available to authorized users.

## Background

Acute lymphoblastic leukemia (ALL) is a malignant disease of the bone marrow characterized by the overproduction of immature white blood cells that accumulate and inhibit the production of normal cells. ALL is the most common type of leukemia in children (Gaynon and Carrel 1999) and major improvements in the treatment of childhood ALL have been achieved in recent years (Pui et al. 2004). However, the treatment outcome remains poor in infant (< 1 year of age) ALL patients due to frequent resistance to cytotoxic chemotherapy drugs, including glucocorticoids (GCs). This condition is associated with a genetic translocation involving the mixed lineage leukemia (MLL) gene (gene KMT2A) that is present in about 80% of infant ALL patients (Greaves 1996; Pieters et al. 2007). Glucocorticoids are used in ALL treatment for their cytotoxicity induction properties that lead to cellular apoptosis (Gaynon and Carrel 1999) and resistance to their effects is the main cause of treatment failure in MLL-rearranged infant ALL (Pieters et al. 1998). Although some researchers have found biomarkers that mediate GC-resistance in MLL-rearranged infant ALL (Spijkers-Hagelstein et al. 2014a; Spijkers-Hagelstein et al. 2013; Spijkers-Hagelstein et al. 2012; Spijkers-Hagelstein et al. 2014b), knowledge regarding the mechanism underlying this phenomenon remains limited. The majority of gene expression studies adopted conventional gene-wise approaches that detect differential expression in each gene separately between two phenotypes.

Motivated by the fact that gene differential co-expression (DC) analysis has emerged as an alternative approach to differential expression analysis (de la Fuente 2010), recently we used weighted gene co-expression network analysis to reveal a gene module associated with GC-resistance (Mousavian et al. 2016) in infant ALL patients. The detected module included genes with documented association to GC-resistance, confirming the hypothesis that network-based analysis complements the conventional gene-wise methods and provides further biological insights into GC-resistance in MLL-rearranged infant ALL. Instead of gene modules, some studies used DC analysis to find phenotype-specific protein modules (Zhang et al. 2012; Lin et al. 2010; Yoon et al. 2011; Chung et al. 2013). Prior to such approach, protein-protein interaction (PPI) networks have been used to find disease-specific protein modules enriched with differentially expressed genes between two groups of samples (Ideker et al. 2002; Chuang et al. 2007; Dittrich et al. 2008; Nacu et al. 2007; Sohler et al. 2004).

In this study, we propose the use of DC analysis to identify protein modules that are active in GC-resistance infant ALL patients but not in GC-sensitive patients. First, gene expression profiles are considered to identify DC gene pairs and construct a DC network between GC-resistance and GC-sensitive conditions. Next, the DC network is modified such that any links that are absent in the experimentally validated PPI network are removed from the DC network. To construct a dynamic protein network for each condition (GC-resistance and GC-sensitive), the resulting network is decomposed into two sub-networks depending on the sign of the difference in co-expression between the two conditions. Finally, each sub-network is clustered into modules. Examining these modules using functional enrichment analysis reveal which of them are highly enriched with gene ontology (GO) terms. Active modules in each condition are specified by extracting genes of modules which are highly enriched in the same category of GO terms and form a connected sub-graph in the corresponding module. Our results demonstrate that protein modules related to signaling pathways such as proteasome, electron transport chain, tRNA-aminoacyl biosynthesis and peroxisome are active under the GC-resistance condition in MLL-rearranged infant ALL patients.

## Methods

### Datasets and preprocessing steps

The infant acute lymphoblastic leukemia gene expression dataset was obtained from the gene expression omnibus (GEO) database under the series accession number GSE32962 (Spijkers-Hagelstein et al. 2012). This dataset consists of expression profiles of 43 untreated infant samples (bone marrow and/or peripheral blood samples) diagnosed with MLL-rearranged ALL and categorized into prednisolone sensitive (19 samples) and prednisolone resistant (24 samples) groups. All leukemic samples contained > 90% of leukemic blasts and contaminating non-leukemic cells were removed using immunomagnetic beads as described in (Kaspers et al. 1994). In vitro prednisolone sensitivity was assessed by 4-day cytotoxicity assays as described in (Pieters et al. 1990). Patient samples were characterized as in vitro sensitive or resistant to prednisolone based on the concentration of prednisolone lethal to 50% of the leukemic cells (LC_50_ value), such that LC_50_ < 0.1 μg/ml of prednisolone indicates prednisolone-sensitive and LC_50_ > 150 μg/ml of prednisolone indicates prednisolone-resistant (Spijkers-Hagelstein et al. 2012). Raw CEL files were downloaded using the *GEOquery* Bioconductor package (Davis and Meltzer 2007). Probe level data was mapped to gene level data using the *Affy* Bioconductor package (Gautier et al. 2004) and the *hgu133plus2.db* Bioconductor human genome annotation package. Intensity levels were normalized using the variance stabilizing normalization method as implemented in the *VSN* Bioconductor package (Huber et al. 2002). The normalized expression matrix consisted of ~ 19,000 rows and 43 columns, representing genes and samples respectively.

The protein-protein interaction data was downloaded from the Human Integrated Protein-Protein Interaction rEference (HIPPIE) database (Schaefer et al. 2012). HIPPIE integrates the experimentally validated PPIs from different sources including BioGrid (Chatr-aryamontri et al. 2013), DIP (Salwinski et al. 2004), HPRD (Prasad et al. 2009), IntAct (Kerrien et al. 2011), MINT (Licata et al. 2012), BIND (Bader et al. 2003) and MIPS (Pagel et al. 2005). The current version includes 203,968 interactions between 14,874 proteins where interactions are given a score between 0 and 1 based on the confidence in used experimental techniques in determining them.

### Construction of DC network and pruning by PPIs

To identify DC gene pairs between GC-sensitive and GC-resistant groups, a DC network was constructed using the *DiffCorr* R package (Fukushima 2013). Pearson’s correlation coefficient was used for calculating the co-expression between gene pairs under conditions *A* (resistant) and *B* (sensitive) separately. Pearson’s correlation coefficient between genes *x* and *y* under condition *A* is defined as

$$ {r}_A\left(x,y\right)=\frac{\sum_{k=1}^{n_A}\left({x}_k-\overline{x}\right)\left({y}_k-\overline{y}\right)}{\sqrt{\sum_{k=1}^{n_A}{\left({x}_k-\overline{x}\right)}^2}\sqrt{\sum_{k=1}^{n_A}{\left({y}_k-\overline{y}\right)}^2}} $$

where $\overline{x}$ and $\overline{y}$ are respectively the mean expressions of gene *x* and *y* under condition *A*, *n*_*A*_ is the number of samples under condition *A*, and *k* is the sample index. The correlation values were transformed using Fisher’s Z transformation such that (Fukushima 2013).

$$ {Z}_A=\frac{1}{2}\log \frac{1+{r}_A}{1-{r}_A} $$

The test statistic for each individual gene pair is the difference between the Z-transformed correlations under conditions *A* and *B* (*Z*_*A*_ and *Z*_*B*_) such that (Fukushima 2013).

$$ Z=\frac{Z_A-{Z}_B}{\sqrt{\frac{1}{n_A-3}+\frac{1}{n_B-3}}} $$

where *n*_*A*_ and *n*_*B*_ are respectively the numbers of samples under conditions *A* and *B*. *DiffCorr* provides a significance of the correlation difference between two conditions (*p*-value) for each individual gene pair and only links with assigned *p*-values< 0.01 are deemed significant and remain in the DC network while the remaining links are removed from the network. The resulting DC network represents links between gene pairs that are differentially co-expressed between GC-resistant and GC-sensitive samples with high confidence. To identify active protein modules, the DC network was modified such that all the links that are absent in the experimentally validated PPI network are removed from the DC network. In other words, the links of the DC network are pruned or trimmed by the experimentally validated protein-protein interactions. Combining the gene expression data and PPI networks in this step is reasonable given the moderate concordance between messenger RNA (mRNA) and protein abundances (Kosti et al. 2016), and provides the bridge between mRNA-based gene expression data and protein modules.

### Decomposing DC network into resistant and sensitive sub-networks

The DC network is further decomposed into two sub-networks, *DC*_*resistant*_ and *DC*_*sensitive*_, based on the weights of the links given by (*r*_*A*_*- r*_*B*_) for individual gene pairs. In the *DC*_*resistant*_ sub-network, only the links that satisfy both of the two conditions *r*_*A*_*- r*_*B*_ > 0.5 and *r*_*A*_ > 0.5 are included. Similarly, in the *DC*_*sensitive*_ sub-network, only links that satisfy both of the two conditions *r*_*A*_*- r*_*B*_ < − 0.5 and *r*_*B*_ > 0.5 are included. These conditions ensure that selected links represent high or moderate co-expression between the respective genes under the condition of interest but not under the second condition. Decomposing the DC network in this way allows easier biological interpretation for detected modules under each condition, as only genes with homogeneous changes between conditions are highlighted in each sub-network.

### Module identification

Two sub-networks *DC*_*resistant*_ and *DC*_*sensitive*_ were constructed to represent active links under each condition. Each sub-network was clustered into modules to identify active protein modules under each condition (resistant and sensitive). We used the generalized version of Topological Overlap Measure (TOM), as implemented in the *WGCNA* R package (Langfelder and Horvath 2008), to define similarity between gene pairs based on the correlation difference. The possible correlation differences can take values between − 2 and 2, and therefore are normalized by a factor of 2 while generating the adjacency matrix of each network. The diagonal elements of the adjacency matrix were set to 1. Then the TOM computes the similarity among gene pairs based on the shared neighbors in the DC networks. The average hierarchical clustering algorithm, as implemented in the *WGCNA* R package (Langfelder and Horvath 2008), was applied to the dissimilarity matrix (1-TOM) to find clusters in each network. The resulting protein modules in each network represent active protein modules under one condition only.

After module identification, we also refined modules in order to maximize the intra-modular connectivity and minimize the inter-modular relationships. A module membership measure was defined for each pair of gene and module based on the connectivity of gene to the corresponding module, and genes were assigned to modules with the highest level of module membership. If a gene is connected to multiple modules with the same number of links, the sum of link weights is used for measuring the module membership value. Genes’ module assignments are iteratively adjusted to reach the optimal assignments.

### Functional enrichment analysis

To determine the potential functions of active protein modules, we imported both *DC*_*resistant*_ and *DC*_*sensitive*_ sub-networks into the *Cytoscape* software platform (Smoot et al. 2011) separately and then used the *BiNGO* application (Maere et al. 2005) to find the overrepresented gene ontology (GO) categories in modules. *BiNGO* uses the hyper-geometric test to determine which gene ontology terms are significantly overrepresented in a module. We also used the Database for Annotation Visualization and Integrated Discovery (DAVID) tool (Dennis Jr et al. 2003) to test if some gene modules are highly enriched with genes from known signaling pathways, including KEGG (Kanehisa et al. 2006) and Reactome (Croft et al. 2010) pathways. GO terms and signaling pathways with FDR corrected *p*-values < 0.01 were deemed significant and selected for describing the functions of different modules.

## Results

### Differential network analysis reveals active protein modules

To detect which gene pairs are differentially co-expressed with significance between resistant and sensitive samples, a weighted differential co-expression network was constructed and only significant links (*p*-value < 0.01) remained in the network. To identify active protein modules, the resulting network was integrated into the PPI network obtained from the HIPPIE database, such that only links available in the PPI network remain. As a result of integrating with the PPI network, a DC network with 4053 links across 3551 nodes (genes or proteins) was obtained. To identify active protein modules in each condition separately, the resulting DC network was decomposed into two *DC*_*resistant*_ and *DC*_*sensitive*_ sub-networks, as described in the Methods section. The *DC*_*resistant*_ sub-network had 1511 links and 1449 nodes, and the *DC*_*sensitive*_ sub-network had 739 links and 1075 nodes.

*DC*_*resistant*_ represents protein links with their associated genes having moderate or high co-expression (correlation) in resistant samples that is also higher than what is observed in sensitive samples. Clustering the *DC*_*resistant*_ sub-network into modules revealed 8 gene modules, with 385 genes, which are active under the resistant condition but not the sensitive condition (see Table 1). Each module was assigned a unique color, and the size of module varies from 20 genes (pink module) to 85 genes (turquoise module). All genes that remained unassigned to any of the 8 modules were placed under the grey module and ignored in this study. Table 1 presents the description of the 8 modules found in the *DC*_*resistant*_ sub-network. The hub gene of each module refers to the gene with highest degree in each module.

**Table 1:** Description of found modules in resistant sub-network

Module	#Genes	#Links	Max/Min Intra-Modular Degree	Hub Gene(s)
Turquoise	85	151	34/1	*PSMC4*
Blue	63	68	38/1	*ELAVL1*
Brown	53	55	15/1	*NDUFA9*
Yellow	46	48	11/1	*LRRK2*
Green	43	44	19/1	*ABCE1*
Red	41	43	10/1	*RARS*, *ATP6V1A*
Violet	34	33	18/1	*APP*
Pink	20	19	14/1	*PEX5*

Applying the same steps to the *DC*_*sensitive*_ sub-network identified 5 modules (see Table 2) comprising 141 genes and ranging in size between 20 (green module) and 39 genes (turquoise module). The remaining unassigned genes were also grouped into the grey module and ignored in further analysis. It is worth stating here that colors were assigned to detected modules in *DC*_*sensitive*_ and *DC*_*resistant*_ independently, i.e. using similar colors for modules in both conditions was totally random. Table 2 provides the description of the 5 detected modules in the *DC*_*sensitive*_ sub-network.

**Table 2:** Description of found modules in sensitive sub-network

Module	#Genes	#Links	Max/Min Intra-Modular Degree	Hub Gene(s)
Turquoise	39	38	11/1	*SIRT7*
Blue	31	30	10/1	*TRAF6*
Brown	27	26	14/1	*NXF1*
Yellow	24	23	17/1	*HNRNPA1*
Green	20	19	17/1	*APP*

### Functional enrichment analysis

After detecting gene modules in both *DC*_*resistant*_ and *DC*_*sensitive*_ sub-networks separately, we performed functional enrichment analysis for all modules using both *BiNGO* and *DAVID* tools. Table 3 lists the significantly enriched biological process (BP) GO terms in the modules of the *DC*_*resistant*_ sub-network. We consider GO terms that are not coarse terms and occupy lower layers of the GO tree. There were no GO terms overrepresented in the blue and violet modules, hence they are not included in Table 3. Table 3 shows that in some modules, such as turquoise, brown, pink and red, a rather large number of module members are involved in the same biological process, hence yielding a high significance (small *p*-value). The turquoise module has ~ 38 out of 85 genes playing key roles in the regulation of protein ubiquitination, ubiquitin-protein ligase activity in mitotic cell cycle, ubiquitin-dependent protein catabolic process and proteolysis, and most of them are highly enriched (FDR = 1.47 × 10^− 48^) in the proteasome KEGG pathway. These genes belong to the proteasome subunit (PSM) family and *PSMC4* with 34 differentially co-expressed links with the rest of the module members is a hub gene in this module. Two other important genes of the same module are *PSMD2* and *PSMD1* with 27 and 22 differentially co-expressed links, respectively. The brown module is highly enriched with some close BP GO terms including mitochondrial adenosine triphosphate (ATP) synthesis coupled electron transport, respiratory electron transport chain and oxidative phosphorylation. Twelve genes of this module are different subunits of NADH:ubiquinone oxidoreductase (complex I), which is the first enzyme complex located in the inner membrane of the mitochondrion and plays a key role in the electron transport chain. In Table 1, *NDUFA9* was introduced as a hub gene of the brown module. *NDUFA9* is differentially co-expressed with other members of the module encoding different subunits of mitochondrial complex I. The brown module was also found significantly enriched with members of the REACTOME Complex I biogenesis and respiratory electron transport pathways. These findings are concordant with earlier studies which associated the up-regulation of oxidative phosphorylation with GC-resistant (Beesley et al. 2009; Samuels et al. 2014).

**Table 3:** Significantly enriched BP GO terms in active protein modules of *DC*_*resistant*_ sub-network

BP GO term	Count	FDR corrected *P*-value
Turquoise module		
positive regulation of ubiquitin-protein ligase activity involved in mitotic cell cycle	33	1.05E-58
negative regulation of ubiquitin-protein ligase activity involved in mitotic cell cycle	32	2.26E-57
anaphase-promoting complex-dependent proteasomal ubiquitin-dependent protein catabolic process	32	3.66E-57
positive regulation of protein ubiquitination	34	1.08E-54
ubiquitin-dependent protein catabolic process	36	9.37E-41
proteolysis	37	3.94E-27
Brown module		
mitochondrial ATP synthesis coupled electron transport	12	1.82E-17
mitochondrial electron transport, NADH to ubiquinone	11	4.66E-17
respiratory electron transport chain	12	5.11E-17
electron transport chain	13	9.51E-16
oxidative phosphorylation	12	9.11E-15
mitochondrion organization	6	1.17E-04
Yellow module		
positive regulation of programmed cell death	9	2.59E-03
MAPKKK cascade	6	2.59E-03
protein amino acid phosphorylation	10	2.59E-03
response to stimulus	24	2.59E-03
programmed cell death	9	3.68E-03
Green module		
RNA splicing	8	6.09E-05
regulation of acetyl-CoA biosynthetic process from pyruvate	3	1.31E-04
spliceosomal snRNP assembly	3	1.61E-03
response to DNA damage stimulus	7	1.80E-03
nuclear mRNA splicing, via spliceosome	4	1.92E-03
glycolysis	3	3.52E-03
Red module		
tRNA aminoacylation	8	1.50E-11
tRNA aminoacylation for protein translation	8	1.50E-11
translation	9	1.88E-06
Pink module		
fatty acid oxidation	6	7.26E-10
peroxisome organization	5	3.55E-09
protein targeting to peroxisome	3	1.37E-05
fatty acid beta-oxidation using acyl-CoA oxidase	2	2.84E-04
acyl-CoA metabolic process	2	1.94E-03
mitochondrion organization	3	4.30E-03

Another module seen in the *DC*_*resistant*_ sub-network is the yellow module. Genes of this module are highly enriched in some distinct BP GO terms including programmed cell death, MAPKKK cascade and response to stimulus. Approximately, 24 genes of the yellow module are highly enriched in response to stimulus and some of these genes are also involved in response to stress. Among 46 genes located in the yellow module, about 10 genes are significantly enriched in programmed cell death and apoptosis. Leucine rich repeat kinase 2 (*LRRK2*) is a gene encoding protein kinase, which is differentially co-expressed with 11 members of the yellow module associated with MAPKKK cascade and also response to stress. Protein kinase C delta (*PRKCD*) is a member of the yellow module that is differentially co-expressed with 9 other members of the module, and is the second hub in the module. Human studies demonstrate that this gene encodes a kinase which is involved in B cell signaling and the regulation of growth and apoptosis.

Some distinct categories of BP GO terms were found in the green module. Respectively, 8 and 7 out of 43 genes were associated with RNA splicing and response to DNA damage. Only 3 genes of this module (*PDHB*, *PDHA1* and *DLAT*) are highly enriched in the acetyl-CoA biosynthetic process, but none of them is a hub gene within the module. Gene *ABCE1* has the highest connectivity within the green module, but *ABCE1* shares no common biological function with its neighbors in the green module.

The red module with 41 genes and 43 DC links is another important module found in the *DC*_*resistant*_ sub-network. Nine out of 39 module members are involved in tRNA aminoacylation for protein translation and are as well members of class I aminoacyl-tRNA synthetase family. The encoded protein by *RARS*, which is a hub gene in this module, belongs to the mentioned protein family and most of its immediate neighbors in the *DC*_*resistant*_ sub-network, including *LARS*, *IARS*, *EPRS*, *DARS* and *MARS*, also encode proteins found in the aminoacyl-tRNA synthetase family. In accordance with this finding, *DAVID* also indicates that the red module is highly enriched in Aminoacyl-tRNA biosynthesis pathway with FDR corrected *p*-value < 10^− 4^.

Although the pink module is the smallest module in the *DC*_*resistant*_ sub-network, it is significantly enriched with more GO terms than some other modules. Enriched terms include fatty acid oxidation, fatty acid catabolic process and peroxisome organization (Table 3). Genes of this module are also highly enriched in Peroxisome KEGG pathway (FDR = 6.07 × 10^− 12^). *PEX5* which plays an essential role in peroxisome, has the highest connectivity within the pink module, and most of its immediate neighbors, including *PEX6*, *ACOX3*, *ACOT8*, *EHHADH*, *HMGCL*, *HACL1*, *ECI2* and *MPV17*, are also involved in Peroxisome KEGG pathway.

**Fig. 1:**
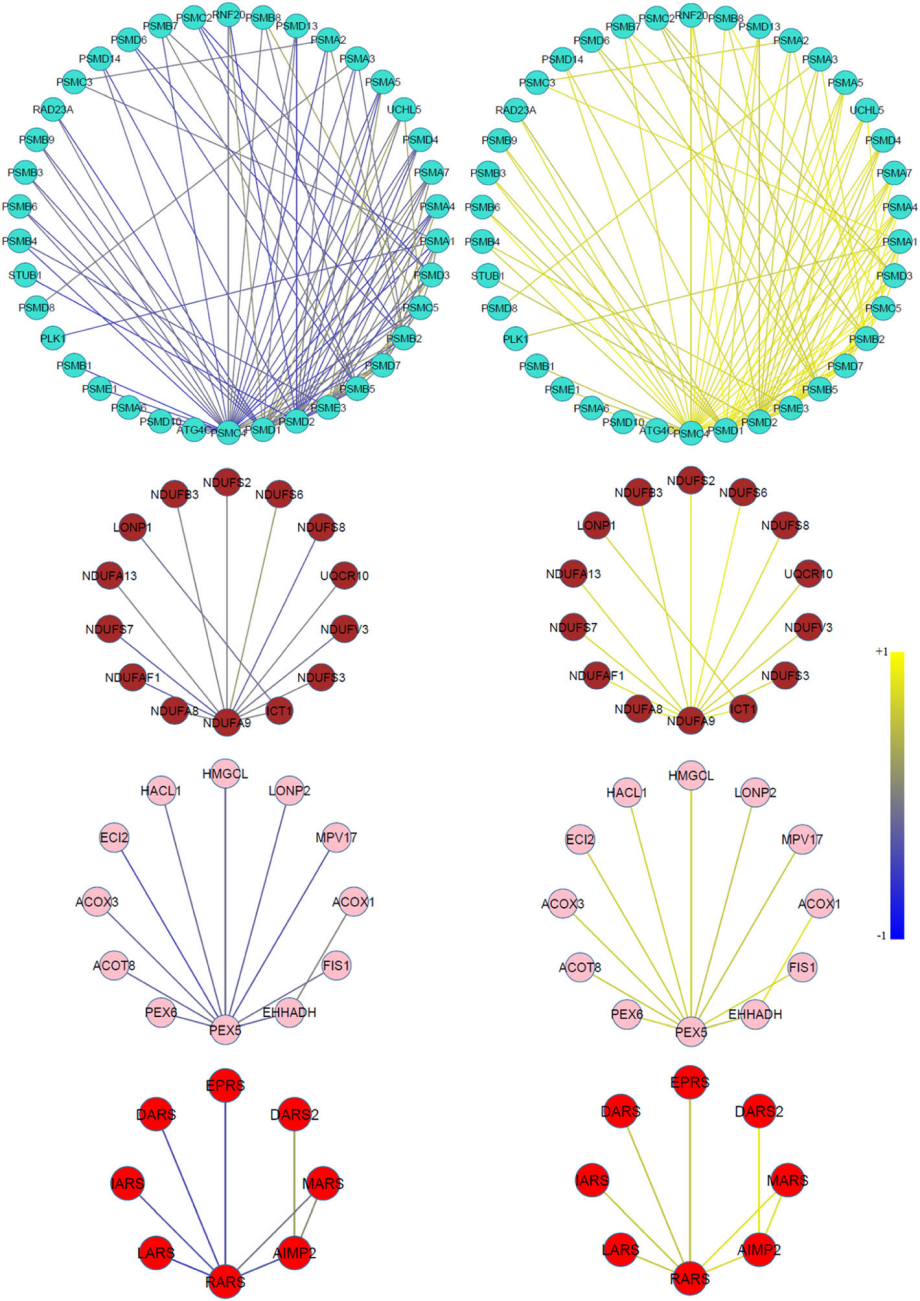
A schematic representation of correlation changes across four protein sub-modules, found in the DC_resistant_ sub-network, between GC-resistance (right side) and GC-sensitive (left side) conditions. Connected genes in the turquoise, brown, pink and red modules are associated with proteasome, respiratory electron transport, peroxisome and aminoacyl-tRNA biosynthesis pathways, respectively, suggesting that the regulatory relationships in these pathways under one condition are disrupted under another. A positive correlation is indicated by yellow color and a negative correlation by blue color

To determine whether the genes associated with biological functions are connected in their corresponding modules, we extract sub-graphs comprising these genes. We observed that the genes associated with proteasome, respiratory electron transport, peroxisome and aminoacyl-tRNA biosynthesis pathways, respectively in the turquoise, brown, pink and red modules are connected within these modules. This indicates that the genes involved in these biological functions are significantly DC between resistant and sensitive condition. This result suggests that the pathways connecting these genes are active pathways where regulatory relationships under one condition are disrupted under another. As indicated in Fig. 1, the links among genes of these pathways are highly co-expressed in the resistant condition in contrast to the sensitive condition.

We performed similar functional enrichment analysis and sub-graph extraction steps to find out whether the detected active modules under the sensitive condition are enriched with BP GO terms. Table 4 shows that only 2 out of 5 modules (blue and yellow modules) of the *DC*_*sensitive*_ sub-network are highly enriched in BP GO terms (FDR < 0.01). In the blue module, 10 genes are involved with protein ubiquitination and proteolysis and ~ 10 genes of the yellow module are involved in RNA splicing and spliceosome KEGG pathway. Although the blue module of *DC*_*sensitive*_ sub-network is involved in proteolysis similar to the turquoise module of *DC*_*resistant*_ sub-network, there are only two genes (*PSMC2* and *PSMD6*) in the intersection of the two modules. Moreover, after extracting the genes associated with proteolysis from the blue module under the sensitive sub-network, no connectivity was observed among them. We also extracted the genes associated with the spliceosome pathway from the yellow module of the *DC*_*sensitive*_ sub-network, and observed that these genes are connected within the module. Hence, the spliceosome can be suggested as an active pathway in sensitive condition as compared to the resistant condition.

**Table 4:** Significantly enriched BP GO terms in active protein modules of *DC*_*sensitive*_ sub-network

BP GO term	Count	FDR corrected *P*-value
Blue module		
proteolysis involved in cellular protein catabolic process	10	9.03E-08
ubiquitin-dependent protein catabolic process	8	5.94E-06
proteolysis	10	8.75E-05
proteasomal ubiquitin-dependent protein catabolic process	5	4.37E-04
protein ubiquitination	5	1.56E-03
myeloid leukocyte activation	3	3.90E-03
positive regulation of ubiquitin-protein ligase activity involved in mitotic cell cycle	3	8.08E-03
Yellow module		
mRNA processing	11	1.74E-11
RNA splicing	10	2.16E-10
nuclear mRNA splicing, via spliceosome	5	5.40E-06

To check the hypothesis that the detected modules in the present study are possibly confounded by differences in prednisolone responsiveness in addition to differences related to GC-resistance, we checked the intersection between the list of 51 transcriptionally-regulated genes by prednisolone reported in (Tissing et al. 2007) and each detected module under the resistant condition. These 51 genes showed differential expression after 8 h of prednisolone exposure in leukemic cells of 13 children as compared with non-exposed cells (Tissing et al. 2007). None of the reported 51 genes appeared in our detected modules under the resistant condition.

## Discussion

Through DC network analysis and protein interaction networks, we identified gene modules which show much higher co-expression under the GC-resistant condition as compared to the GC-sensitive condition. After detecting gene modules from the integration of DC network between GC-sensitive and GC-resistant samples and PPI links, functional enrichment analysis detected which members of modules share similar biological functions or are members of the same biological pathway. Together, these results suggest that four gene sub-modules, obtained from the turquoise, brown, pink and red modules of DC_resistant_ sub-network, are respectively associated with the proteasome, mitochondrial respiratory electron transport, peroxisome and aminoacyl-tRNA biosynthesis signaling pathways (Fig. 1). The lists of genes (ranked by the inter-modular connectivity) present in these four modules are given in Additional file 1: Tables S1-S4. The yellow module was identified in the DC_resistant_ sub-network as a significantly enriched module in the immune system process, and this module shares 11 genes with a module we found in our previous study (Mousavian et al. 2016) which was introduced as a relevance module to GC-resistant in infant ALL.

In 1993, the proteasome has been localized with high serum concentration in tumor cells of patients with hematological malignancy (Ichihara 1993). It was found later that NF-κB (nuclear factor kappa-light-chain-enhancer of activated B cells) can mediate glucocorticoid resistance in multiple myeloma, which is a cancer formed by terminally differentiated B Cells (Feinman et al. 1999; Tricot 2002). NF-κB is a heterodimeric transcription factor that activates survival genes coding for cytokines, cytokine receptors, chemotactic proteins and cell adhesion molecules (De Bosscher et al. 2000) and repressing its transcriptional activity facilitates cellular apoptosis. In many cell types, the function of NF-κB depends on the enzymatic activity of proteasome (Baud and Derudder 2010). Through the degradation of the inhibitory protein, I-κBα, protein subunit of NF-κB including RELA or c-Rel is allowed to activate expression of target genes after entering the nuclease. In 2003, Bortezomib (a proteasome inhibitor) was found efficient in treating patients whose multiple myeloma showed poor response to at least two treatment protocols (Dick and Fleming 2010; Lambrou et al. 2012). In recent years, the effectiveness of Bortezomib was also tested for the treatment of acute lymphoblastic leukemia. It was shown that bortezomib can sensitize in-vitro GC-resistant childhood B-cell precursor leukemia cell lines, MHH-cALL-2 and MHH-cALL-3, to prednisolone-induced cell death via inhibiting the proteasome (Junk et al. 2015). The use of proteasome inhibitors to sensitize GC-resistant ALL cells was supported by detecting that high expression of valosin-containing protein (VCP), a member of the ubiquitin proteasome degradation system (UPS), is associated with poor response to prednisolone treatment in childhood ALL patients (Lauten et al. 2006). Valosin-containing protein mediates apoptosis after tumor necrosis factor (TNF) stimulation by influencing the proteasome degradation pathway and NF-κB activation via I-κBα degradation (Asai et al. 2002). The immunosuppressive effects of glucocorticoids are linked to an inhibition of NF-κB activity (Greenstein et al. 2002; Scheinman et al. 1995; Auphan et al. 1995), suggesting that suppressing the NF-κB activity is required for glucocorticoid-induced apoptosis (Chandra et al. 1998). Our results show that the activity of proteasome and ubiquitination family genes (enriched in the turquoise module) is significantly higher in GC-resistant MLL-rearranged infant ALL patients as compared to GC-sensitive patients. Our results agree with related literature in suggesting that inhibiting the proteasome protein family members, which are crucial in regulating protein ubiquitination and proteasome pathway, may lead to sensitizing the infant ALL cells to prednisolone.

In the DC_resistant_ sub-network, we found a set of genes related to the NADH:ubiquinone oxidoreductase activity, which forms complex I in mitochondria for electron transport chain, as a differential co-expressed gene set between GC-resistant and GC-sensitive samples. Some studies demonstrated that GC-resistance in T-cell ALL is associated with a proliferative metabolism such as the up-regulation of glycolysis, oxidative phosphorylation and cholesterol biosynthesis (Beesley et al. 2009; Samuels et al. 2014). It was shown that the activation of bioenergetic pathways required for proliferation may suppress the apoptotic potential and offset the metabolic crisis initiated by glucocorticoids in the lymphocytes (Beesley et al. 2009). It was also shown later that targeting bioenergetic pathways in combination with glucocorticoid treatment may offer a promising therapeutic strategy to overcome GC-resistance in ALL (Samuels et al. 2014). The detected brown module has 13 genes that are involved in oxidative phosphorylation which is a metabolic pathway for oxidizing nutrients and releasing energy in the mitochondria. These genes are highly co-expressed in GC-resistant infant ALL in comparison with the sensitive cases, indicating that GC-resistance in infant ALL may also be associated with some proliferative metabolism like oxidative phosphorylation. High expression of the valosin-containing protein, that mediates NF-κB activation via I-κBα degradation, is associated with poor response (resistance) to prednisolone treatment in childhood ALL patients as discussed above (Lauten et al. 2006). NF-κB acts through the transcription of anti-apoptotic proteins, leading to increased proliferation and growth activities (Escarcega et al. 2007). Therefore, detecting an increased co-expression between genes associated with proliferation and oxidative phosphorylation in GC-resistant ALL infants might (at least in part) be explained through this mechanism.

Another important active protein module observed in DC_resistant_ sub-network is the pink module. The pink module contains genes involved in fatty acid oxidation and peroxisome organization. The peroxisome is a small cell organelle which contributes to the breakdown of very-long-chain fatty acids via beta oxidation. Recently, it was indicated that the peroxisome proliferator-activated receptor alpha (PPARα) and fatty acid oxidation mediate glucocorticoid resistance in chronic lymphoblastic leukemia (CLL) (Tung et al. 2013). Gene *PEX5* (Peroxisomal Biogenesis Factor 5), the hub gene of the pink module detected in the resistant sub-network, is associated with fatty acid beta oxidation, peroxisome pathway, and glucose metabolism. Also gene *ABCE1* (ATP Binding Cassette Subfamily E Member 1), the hub gene of the green module detected in the resistant sub-network, is associated with glucose transport. The recent work by Chan et al. (Chan et al. 2017) characterized pre-B-cell ALL with transcriptional repression of glucose and energy supply. Chan et al. found that the *PAX5* and *IKZF1* B-lymphoid transcription factors enforce a state of chronic energy deprivation in pre-B-cell ALL cells, and identified, among others, products of gene *NR3C1* (a transcription factor gene encoding the glucocorticoid receptor that bind to glucocorticoid response elements and activate their transcription) as central effectors of B-lymphoid restriction of glucose and energy supply. More specific to MLL-rearranged infant ALL, the data reported in (Stumpel et al. 2009) independently showed that *NR3C1* is among the top 100 genes with significant hypermethylated promoter region in t(4;11)-positive MLL-rearranged infant ALL samples. Hence, the literature already presents possible mechanisms (transcriptional targets and promoter methylation) by which glucose metabolism alterations and energy deprivation could be associated with MLL-rearranged infant ALL cells. Although Chan et al. did not conduct their study using MLL-rearranged infant samples, the similarities between their findings and the current study are within the general characterization of B-cell ALL with transcriptional repression of glucose metabolism and energy supply. In addition to the pink module, the brown module found in resistant sub-network is rich with genes related to ATP synthesis by chemiosmotic coupling, adding additional indication to energy deprivation in B-cell ALL.

Our results indicate that the red module is highly associated with the Aminoacyl-tRNA biosynthesis pathway where 9 of its 39 module members belong to the Aminoacyl-tRNA synthetases family. Aminoacyl-tRNA synthetases (ARSs) are essential house-keeping enzymes that provide the substrates for protein synthesis (Yao and Fox 2013). They have been implicated with human cancers, given their varied effects on cell differentiation and growth. It was discovered since the 1960s that leukemic blasts require external asparagine (an ARS) for growth since they lack sufficient activity of asparagine synthetase. A component of guinea pig serum, L-asparaginase, was isolated and successfully used to convert free asparagine to aspartic acid, effectively starving the leukemia cells (Broome 1963). L-asparaginase has been used as a component of the chemotherapy in the treatment of childhood ALL in combination with glucocorticoids (prednisolone and dexamethasone), and vincristine (Dübbers et al. 1998). Similar to glucocorticoids, some patients show resistance to L-asparaginase and in vitro resistance was found highly correlated with an increase in the cellular asparagine synthetase activity, messenger RNA and protein content (Hutson et al. 1997). On another vein, the expression of glutamyl-prolyl-tRNA synthetase and mitochondrial isoleucyl-tRNA synthetase is controlled by the c-*myc* proto-oncogene (Coller et al. 2000), hence abnormal expression of these tRNA synthetases under oncogenic conditions is not surprising. In addition to NF-κB and activator protein 1 (AP-1), c-*myc* was one of three transcription factors identified as the most likely targets of GC-induced gene repression (Greenstein et al. 2002). Previous studies have revealed correlations between c-*myc* suppression and GC-induced apoptosis in human leukemic cells (Thulasi et al. 1993). Interestingly, the transforming growth factor-β (TGF-β) induces nuclear localization of the aminoacyl-tRNA synthetase-interacting factor 2 (AIMP2), where AIMP2 enhances ubiquitin-dependent degradation of the FUSE-binding protein (FBP), which is the transcriptional activator of c-*myc* (Kim et al. 2003), resulting in down-regulation of c-*myc*. Detecting the red module that is highly enriched with ARSs in GC-resistant patients may indicate failure to repress c-*myc* and initiate GC-induced apoptosis due to increased cellular activity of ARS interacting factors. Additionally, a few mutations of the aminoacyl-tRNA synthetase-interacting factor 3 (AIMP3) that affect its interaction with ataxia-telangiectasia mutated (ATM) kinases and ability to activate p53 (a tumor suppressor protein) have been reported in human chronic myeloid leukemia patients (Kim et al. 2008). These observations further support the relationship between ARSs and/or their interacting factors with the initiation or progression of human leukemia.

## Conclusion

Differential co-expression analysis is a promising approach to incorporate the dynamic context of gene expression profiles into experimentally-validated protein-protein interaction networks. The approach allows the detection of relevant gene modules that are highly enriched with DC gene pairs and reduces the problem of detecting modules of co-expressed genes that are not truly related by discarding all gene-pairs not documented in the PPI databases. Functional enrichment analysis of detected modules revealed that these modules are related to proteasome, electron transport chain, tRNA-aminoacyl biosynthesis, and peroxisome signaling pathways. These findings are in accordance with reported literature related to GC-resistance in hematological malignancies such as pediatric ALL. Our results support the use of proteasome inhibitors and asparagine depletion drugs as components of the chemotherapy in the treatment of childhood ALL for patients showing resistance to glucocorticoids. Our results also support the characterization of B-cell ALL with chronic glucose metabolism and energy supply deprivation.

## Additional file

Additional file 1: **Table S1.** The list of genes present in the turquoise module. **Table S2** The list of genes present in the pink module. **Table S3.** The list of genes present in the brown module. **Table S4.** The list of genes present in the red module. (DOCX 11 kb)

## Data Availability

The datasets generated during and/or analyzed during the current study are available from the corresponding author on reasonable request.

## Abbreviations

ALL: Acute Lymphoblastic leukemia
ARS: Aminoacyl-tRNA synthetase
ATM: Ataxia-Telangiectasia Mutated
ATP: Adenosine triphosphate
BP: Biological process
CLL: Chronic lymphoblastic leukemia
DC: Differential co-expression
FDR: False discovery rate
GCs: Glucocorticoids
GEO: Gene expression omnibus
GO: Gene ontology
MLL: Mixed lineage leukemia
PPI: Protein-protein interaction
TOM: Topological Overlap Measure
