# Supplementary material for: Differential network analysis and protein-protein interaction study reveals active protein modules in glucocorticoid resistance for infant acute lymphoblastic leukemia
Source: Mol Med. 2019 Aug 1;25:36. doi: 10.1186/s10020-019-0106-1 (PMC6676637; doi:10.1186/s10020-019-0106-1)
Supplement: Supplementary file 1 — Table S1. The list of genes present in the turquoise module. Table S2 The list of genes present in the pink module. Table S3. The list of genes present in the brown module. Table S4. The list of genes present in the red module. (DOCX 11 kb) [file 10020_2019_106_MOESM1_ESM.docx]

| **Gene** | **#Inter-modular Connectivity** |
| --- | --- |
| PSMC4 | 27 |
| PSMD1 | 17 |
| PSMD2 | 15 |
| PSME3 | 10 |
| PSMB5 | 9 |
| PSMD7 | 8 |
| PSMB2 | 8 |
| PSMA1 | 7 |
| PSMC5 | 7 |
| PSMD3 | 7 |
| PSMA7 | 5 |
| PSMA4 | 5 |
| PSMA3 | 4 |
| UCHL5 | 4 |
| PSMD4 | 4 |
| PSMA2 | 4 |
| PSMA5 | 4 |
| RNF20 | 3 |
| PSMD13 | 3 |
| PSMC2 | 3 |
| PSMB7 | 3 |
| PSMB8 | 3 |
| RAD23A | 2 |
| PSMC3 | 2 |
| PSMB6 | 2 |
| PSMB3 | 2 |
| PSMB9 | 2 |
| PSMD14 | 2 |
| PSMB4 | 2 |
| PSMD6 | 2 |
| PSMD8 | 1 |
| STUB1 | 1 |
| PSMD10 | 1 |
| ATG4C | 1 |
| PSMA6 | 1 |
| PSME1 | 1 |
| PSMB1 | 1 |
| PLK1 | 1 |

**Table S1** The list of genes present in the turquoise module

**Table S2** The list of genes present in the pink module

| **Gene** | **#Inter-modular Connectivity** |
| --- | --- |
| PEX5 | 11 |
| EHHADH | 2 |
| MPV17 | 1 |
| LONP2 | 1 |
| HMGCL | 1 |
| HACL1 | 1 |
| ECI2 | 1 |
| ACOX3 | 1 |
| ACOX1 | 1 |
| ACOT8 | 1 |
| PEX6 | 1 |
| FIS1 | 1 |

**Table S3** The list of genes present in the brown module

| **Gene** | **#Inter-modular Connectivity** |
| --- | --- |
| NDUFA9 | 11 |
| ICT1 | 2 |
| NDUFS3 | 1 |
| NDUFV3 | 1 |
| NDUFS7 | 1 |
| UQCR10 | 1 |
| NDUFAF1 | 1 |
| NDUFS8 | 1 |
| NDUFA8 | 1 |
| NDUFS2 | 1 |
| NDUFB3 | 1 |
| NDUFA13 | 1 |
| LONP1 | 1 |
| NDUFS6 | 1 |
| DAP3 | 1 |
| NDUFB6 | 1 |
| SSBP1 | 1 |

**Table S4** The list of genes present in the red module

| **Gene** | **# Inter-modular Connectivity** |
| --- | --- |
| RARS | 6 |
| AIMP2 | 3 |
| MARS | 2 |
| WARS | 2 |
| DARS2 | 1 |
| DARS | 1 |
| LARS | 1 |
| IARS | 1 |
| EPRS | 1 |
